# Supplementary material for: Quantum frequency doubling in the topological insulator Bi2Se3
Source: Nat Commun. 2021 Jan 29;12:698. doi: 10.1038/s41467-021-20983-1 (PMC7846578; doi:10.1038/s41467-021-20983-1)
Supplement: Supplementary file 1 — Supplementary Information [file 41467_2021_20983_MOESM1_ESM.pdf]

SUPPLEMENTARY INFORMATION

Quantum frequency doubling in the topological insulator Bi_2Se_3

Pan He[1,2†], Hiroki Isobe[3†], Dapeng Zhu[1], Chuang-Han Hsu[1], Liang Fu[3*] and Hyunsoo Yang[1*]

[1]*Department of Electrical and Computer Engineering, National University of Singapore, 117576, Singapore*

[2]*Institute for Nanoelectronic devices and Quantum computing, Fudan University, Shanghai 200433, China*

[3]*Department of Physics, Massachusetts Institute of Technology, Cambridge, Massachusetts 02139, USA*

[†]These authors contributed equally to this work. *e-mail: liangfu@mit.edu; eleyang@nus.edu.sg

Supplementary Note 1. Nonlinear electric transport and second-harmonic generation (SHG)

Nonlinear electric transport suggests that the transverse current density J_y under an applied longitudinal electric field E_x has a nonlinear response such as $\sigma_{yxx}^{(2)}E_x^2$, in addition to the conventional linear one $\sigma_{yx}^{(1)}E_x$:

$$J_y=\sigma_{yx}^{(1)}E_x+\sigma_{yxx}^{(2)}E_x^2. \quad (1)$$

The conductivity $\sigma_{yx}=\frac{J_y}{E_x}=\sigma_{yx}^{(1)}+\sigma_{yxx}^{(2)}E_x$ is thus electric field dependent. Under time reversal symmetry, the linear conductivity $\sigma_{yx}^{(1)}$ vanishes, and the transverse resistivity ρ_{yx} (considering $\sigma_{yx}=\frac{-\rho_{yx}}{\rho_{xx}^2+\rho_{yx}^2}, \rho_{xx}^2 >> \rho_{yx}^2$) can be expressed as a function of current I_x as

$$\rho_{yx}\approx-\rho_{xx}^2\cdot\sigma_{yx}=-\rho_{xx}^2\cdot\sigma_{yxx}^{(2)}\cdot E_x=-\rho_{xx}^2\cdot\sigma_{yxx}^{(2)}\cdot R_{xx}\cdot I_x/L \quad (2)$$

where L is the length of Hall bar. For 3D TI, only the surface can host nonlinear electric transport according to the symmetry analysis. The transverse resistance

$$R_{yx}=\frac{V_y}{I_x}=\frac{E_y\cdot W}{J_x\cdot W}=\rho_{yx}=-\rho_{xx}^2\cdot\sigma_{yxx}^{(2)}\cdot R_{xx}\cdot I_x/L,$$

where W is the width of Hall bar. Thus, $R_{yxx}^{(2)}\equiv\frac{R_{yx}}{I_x}=\frac{V_y}{I_x^2}=-\rho_{xx}^2\cdot\sigma_{yxx}^{(2)}\cdot R_{xx}/L$, proportional to nonlinear conductivity $\sigma_{yxx}^{(2)}$, can be used to quantify the nonlinear transverse transport.

To extract the nonlinear transverse voltage $V_y=R_{yxx}^{(2)}\cdot I_x^2$, which scales quadratically with the applied current, harmonic measurements can be used. When a sinusoidal ac current $I_\omega=I\sin\omega t$ is applied to the device, the transverse voltage

$$V_y=R_{yxx}^{(2)}I^2\sin^2\omega t=\frac{1}{2}R_{yxx}^{(2)}I^2+\frac{1}{2}R_{yxx}^{(2)}I^2\sin(2\omega t-\pi/2), \quad (3)$$

consists of a dc, and an out-of-phase SHG term. The SHG amplitude $V_y^{2\omega}=\frac{1}{2}R_{yxx}^{(2)}I_x^2$ is exactly half of the nonlinear transverse voltage. Thus, we can explore the nonlinear electric transport by measuring the SHG using the lock-in technique with a high sensitivity.

Supplementary Note 2. Skew scattering by screened Coulomb impurities and the Berry curvature triple

The Hamiltonian for the TI surface state is $H_{\mathrm{TI}} = \hbar v(k_x\sigma_y - k_y\sigma_x) + \frac{\lambda}{2}(k_+^3 + k_-^3)\sigma_z$ with $k_\pm = k_x \pm ik_y$. When we focus the conduction band and write the eigenstate as $|\boldsymbol{k}\rangle$, the energy dispersion is $\epsilon_{\boldsymbol{k}} = \sqrt{\hbar^2v^2k^2 + \lambda^2k^6\cos 3\theta_{\boldsymbol{k}}}$, and the z component of the Berry curvature is $\Omega_z(\boldsymbol{k}) = \frac{\lambda}{\hbar|v|}\cos 3\theta_{\boldsymbol{k}} + O(\lambda^2)$ with $\theta_{\boldsymbol{k}} = \arg\boldsymbol{k}$.

The bare Coulomb interaction with a charge carrier and an impurity with charge Q is $V_0(r) = \frac{e^2Q}{4\pi\varepsilon_0\varepsilon r}$, where ε_0 is the vacuum permittivity and ε is the dielectric constant that depends on the environment adjacent to the TI surface. We introduce the dimensionless coupling constant analogous to the fine-structure constant: $\alpha \equiv \frac{e^2Q}{4\pi\varepsilon_0\varepsilon\hbar v}$. Then, we consider the Fourier transform of the Coulomb interaction $V_0(q) = \frac{2\pi\alpha\hbar v}{q}$. With the Thomas-Fermi screening at long wavelength, the screened Coulomb interaction takes the form $V(q) = \frac{2\pi\alpha\hbar v}{q+q_{\mathrm{TF}}}$. Here, q_{TF} is the Thomas-Fermi wave vector given at zero temperature: $q_{\mathrm{TF}} = 2\pi\alpha D(\epsilon_F)$, which is proportional to the density of states at the Fermi energy $D(\epsilon_F) = \frac{\epsilon_F}{2\pi\hbar^2v^2} + O(\lambda^2)$. We also obtain the matrix element of the screened Coulomb interaction on the Fermi surface: $V_{\boldsymbol{k}\boldsymbol{k}'} = \int dr\, e^{-i(\boldsymbol{k}-\boldsymbol{k}')\cdot\boldsymbol{r}}\langle\boldsymbol{k}|V(r)|\boldsymbol{k}'\rangle = \frac{2\pi\alpha\hbar v}{|\boldsymbol{k}-\boldsymbol{k}'|+q_{\mathrm{TF}}}\langle\boldsymbol{k}|\boldsymbol{k}'\rangle = \frac{2\pi\alpha\hbar v}{k_F\left|\sin\left(\frac{\theta_{\boldsymbol{k}}-\theta_{\boldsymbol{k}'}}{2}\right)\right|+q_{\mathrm{TF}}}\left[\frac{1+e^{-i(\theta_{\boldsymbol{k}}-\theta_{\boldsymbol{k}'})}}{2} + \frac{\lambda k_F^2}{4\hbar|v|}\left(1 - e^{-i(\theta_{\boldsymbol{k}}-\theta_{\boldsymbol{k}'})}\right)(\cos 3\theta_{\boldsymbol{k}} + \cos 3\theta_{\boldsymbol{k}'}) + O(\lambda^2)\right]$, where k_F is the Fermi wavevector.

For a semiclassical Boltzmann analysis, we need the scattering rate $w_{\boldsymbol{k}\boldsymbol{k}'}$, which describes the transition rate from the Bloch state with $\boldsymbol{k}$ to that with $\boldsymbol{k}'$. The second-order response can only be finite in a noncentrosymmetric medium, so we decompose the scattering rate into symmetric and antisymmetric parts under inversion: $w_{\boldsymbol{k}\boldsymbol{k}'}^{(S)} = \frac{1}{2}(w_{\boldsymbol{k}\boldsymbol{k}'} + w_{-\boldsymbol{k},-\boldsymbol{k}'})$, $w_{\boldsymbol{k}\boldsymbol{k}'}^{(A)} = \frac{1}{2}(w_{\boldsymbol{k}\boldsymbol{k}'} - w_{-\boldsymbol{k},-\boldsymbol{k}'})$. Note that time-reversal symmetry guarantees $w_{\boldsymbol{k}\boldsymbol{k}'} = w_{-\boldsymbol{k}',-\boldsymbol{k}}$. The lowest order contributions to $w_{\boldsymbol{k}\boldsymbol{k}'}^{(S)}$ and $w_{\boldsymbol{k}\boldsymbol{k}'}^{(A)}$ in perturbative calculations about the impurity potential appear at second and third orders, respectively: $w_{\boldsymbol{k}\boldsymbol{k}'}^{(S)} = 2\pi\langle|V_{\boldsymbol{k}\boldsymbol{k}'}|^2\rangle_{\mathrm{dis}}\delta(\epsilon_{\boldsymbol{k}} - \epsilon_{\boldsymbol{k}'}) = 2\pi n_i|V_{\boldsymbol{k}\boldsymbol{k}'}|^2\delta(\epsilon_{\boldsymbol{k}} - \epsilon_{\boldsymbol{k}'})$, $w_{\boldsymbol{k}\boldsymbol{k}'}^{(A)} =$

$-(2\pi)^2 \int \frac{d^2q}{(2\pi)^2} \mathrm{Im}\langle V_{\boldsymbol{k}'\boldsymbol{q}} V_{\boldsymbol{q}\boldsymbol{k}} V_{\boldsymbol{k}\boldsymbol{k}'} \rangle_{\mathrm{dis}} \delta(\epsilon_{\boldsymbol{k}} - \epsilon_{\boldsymbol{k}'}) \delta(\epsilon_{\boldsymbol{k}'} - \epsilon_{\boldsymbol{q}}) =$

$-(2\pi)^2 n_i \int \frac{d^2q}{(2\pi)^2} \mathrm{Im}[V_{\boldsymbol{k}'\boldsymbol{q}} V_{\boldsymbol{q}\boldsymbol{k}} V_{\boldsymbol{k}\boldsymbol{k}'}] \delta(\epsilon_{\boldsymbol{k}} - \epsilon_{\boldsymbol{k}'}) \delta(\epsilon_{\boldsymbol{k}'} - \epsilon_{\boldsymbol{q}})$. Here, we assume that impurities are randomly distributed in the sample with the density n_i, where $\langle\ \rangle_{\mathrm{dis}}$ denotes the impurity average. We also assume low impurity concentration and expand disorder averages with respect to the impurity density n_i to keep the leading-order contributions.

For the screened Coulomb interaction, we obtain the symmetric part of the scattering rate as $w_{\boldsymbol{k}\boldsymbol{k}'}^{(S)} = \frac{4\pi^3 n_i \alpha^2 \hbar^2 v^2}{(|\boldsymbol{k}-\boldsymbol{k}'|+q_{\mathrm{TF}})^2} (1 + \hat{\boldsymbol{k}} \cdot \hat{\boldsymbol{k}}') \delta(\epsilon_{\boldsymbol{k}} - \epsilon_{\boldsymbol{k}'}) + O(\lambda)$, where $\hat{\boldsymbol{k}} = \boldsymbol{k}/|\boldsymbol{k}|$ denotes the unit vector. It leads to the scattering time $\tau^{-1} = \int \frac{d^2k'}{(2\pi)^2} w_{\boldsymbol{k}\boldsymbol{k}'}^{(S)} = \frac{\pi^2 n_i \alpha^2 \hbar v}{k_F}$ for $q_{\mathrm{TF}}/k_F \lesssim 1$. We can also obtain the solution for $q_{\mathrm{TF}}/k_F \gg 1$, but we should instead consider short-range impurities of the form $\delta(\boldsymbol{r})$, which is not the focus here. For the antisymmetric part, the contribution appears from order λ and we only consider to the order perturbatively. Since it respects threefold rotational symmetry, we expand it in a harmonic series and we find the largest contribution takes the form $\cos 3\theta_{\boldsymbol{k}} + \cos 3\theta_{\boldsymbol{k}'}$. From this observation, we may deduce that $w_{\boldsymbol{k}\boldsymbol{k}'}^{(A)}$ is related to the Berry curvature $\Omega_z(\boldsymbol{k})$ to obtain $w_{\boldsymbol{k}\boldsymbol{k}'}^{(A)} \simeq -\pi^4 n_i \alpha^3 \hbar^2 v^2 c_1\left(\frac{q_{\mathrm{TF}}}{k_F}\right) [\Omega_z(\boldsymbol{k}) + \Omega_z(\boldsymbol{k}')] \frac{\hat{z}\cdot(\hat{\boldsymbol{k}}\times\hat{\boldsymbol{k}}')}{|\hat{\boldsymbol{k}}-\hat{\boldsymbol{k}}'|+\frac{q_{\mathrm{TF}}}{k_F}} \delta(\epsilon_{\boldsymbol{k}} - \epsilon_{\boldsymbol{k}'})$, where c_1 is a coefficient given by $c_1(q_s) = \int \frac{d\theta_{\boldsymbol{k}}}{2\pi} \frac{d\theta_{\boldsymbol{k}'}}{2\pi} (\cos 3\theta_{\boldsymbol{k}} + \cos 3\theta_{\boldsymbol{k}'}) F_1(\theta_{\boldsymbol{k}}, \theta_{\boldsymbol{k}'}; q_s)$ with $F_1(\theta_{\boldsymbol{k}}, \theta_{\boldsymbol{k}'}; q_s) = 4 \int \frac{d\theta_{\boldsymbol{q}}}{2\pi} \frac{1}{2\left|\sin\frac{\theta_{\boldsymbol{q}}-\theta_{\boldsymbol{k}'}}{2}\right|+q_s} \frac{1}{2\left|\sin\frac{\theta_{\boldsymbol{k}}-\theta_{\boldsymbol{q}}}{2}\right|+q_s} \times \left[\frac{\cos 3\theta_{\boldsymbol{k}} \sin(\theta_{\boldsymbol{q}}-\theta_{\boldsymbol{k}'}) + \cos 3\theta_{\boldsymbol{k}'} \sin(\theta_{\boldsymbol{k}}-\theta_{\boldsymbol{q}})}{\sin(\theta_{\boldsymbol{k}}-\theta_{\boldsymbol{k}'})} - \cos 3\theta_{\boldsymbol{q}}\right]$. The numerical result of c_1 is shown in Supplementary Fig. 5, which decays for large q_{TF}/k_F. We estimate the carrier density of the topological surface state $n_{\mathrm{TSS}} = 2.43 \times 10^{12}\ \mathrm{cm}^{-2}$ corresponds to the Fermi wavelength $\lambda_F = \sqrt{\pi/n_{\mathrm{TSS}}} = 11.4$ nm and the Thomas-Fermi screening length of the Bi_2Se_3 surface state ranges from 26 to 90 nm[1,2] to find the ratio $q_{\mathrm{TF}}/k_F \lesssim 0.4$.

It is worth pointing out the directional dependence of the scattering rates $w_{\boldsymbol{k}\boldsymbol{k}'}^{(S)}$ and $w_{\boldsymbol{k}\boldsymbol{k}'}^{(A)}$: The former contains the form factor $(1 + \hat{\boldsymbol{k}} \cdot \hat{\boldsymbol{k}}')$ and the latter $\hat{z} \cdot (\hat{\boldsymbol{k}} \times \hat{\boldsymbol{k}}')$, which manifests skew scattering. Those form factors, on the other hand, do not reflect the threefold rotational symmetry of the model; it appears in $w_{\boldsymbol{k}\boldsymbol{k}'}^{(A)}$ through the Berry curvature $\Omega_z(\boldsymbol{k})$. Therefore, the magnitude and the $\boldsymbol{k}$ dependence of the Berry curvature control the strength of skew scattering. To quantify it, we

introduce the *Berry curvature triple* T as a convolution of the Berry curvature and the angular form factor for threefold rotation symmetry: $T(\epsilon_F) = 2\pi\hbar \int \frac{d^2k}{(2\pi)^2} \delta(\epsilon_F - \epsilon_{\boldsymbol{k}}) \Omega_z(\boldsymbol{k}) \cos 3\theta_{\boldsymbol{k}}$, which has a dimension of time. For the TI surface model, the Berry curvature triple is given by $T(\epsilon_F) = \frac{\pi\lambda}{|v|} D(\epsilon_F) + O(\lambda^2)$, which is a product of the density of states and the Berry curvature on the Fermi surface, reflecting the threefold rotational symmetry of the model.

Since the angular dependence $\hat{z} \cdot (\hat{\boldsymbol{k}} \times \hat{\boldsymbol{k}}')$ typically represents skew scattering, we may characterize the strength of skew scattering by writing the antisymmetric part of the scattering rate as $w_{\boldsymbol{kk}'}^{(A)} \equiv \widetilde{w}_{\boldsymbol{kk}'}^{(A)} \hat{z} \cdot (\hat{\boldsymbol{k}} \times \hat{\boldsymbol{k}}')$ and we can approximate the skew scattering time $\tilde{\tau}$ for $q_{\mathrm{TF}}/k_F \lesssim 1$ by $\tilde{\tau}^{-1} \approx \int \frac{d^2k}{(2\pi)^2} \widetilde{w}_{\boldsymbol{kk}'}^{(A)} \approx 4\pi^2 n_i \alpha^3 v^2 c_1 \left(\frac{q_{\mathrm{TF}}}{k_F}\right) T(\epsilon_F)$. Here the numerical factor is determined to match the result for unscreened Coulomb impurities $\tilde{\tau}^{-1} \approx 2\pi^2 n_i \lambda \alpha^3 k_F/\hbar$.[3] This equation shows the relation between the skew scattering time and the Berry curvature triple in a threefold rotational symmetric system.

One may argue the density dependence of the second-order response from skew scattering using the expression $\sigma^{(2)} = \frac{e^3 v \tau^3}{\hbar^2 \tilde{\tau}}$. For $q_{\mathrm{TF}}/k_F \lesssim 1$, we find the density dependence of the second-order conductivity $\sigma^{(2)} \propto k_F^4 \propto n_{\mathrm{TSS}}^2$, which grows quadratically as the carrier density increases. At high densities, however, stronger screening of the Coulomb interaction weakens the second-order response. Higher-order effects in terms of the warping λ become stronger. Moreover, the bulk states also contribute to screening and interfere the isolated surface states. Recall that the bulk remains inversion symmetric. In summary, one expects that $\sigma^{(2)}$ increases as n_{TSS}^2 at low densities and decreases at high densities due to stronger screening effects and the bulk states.

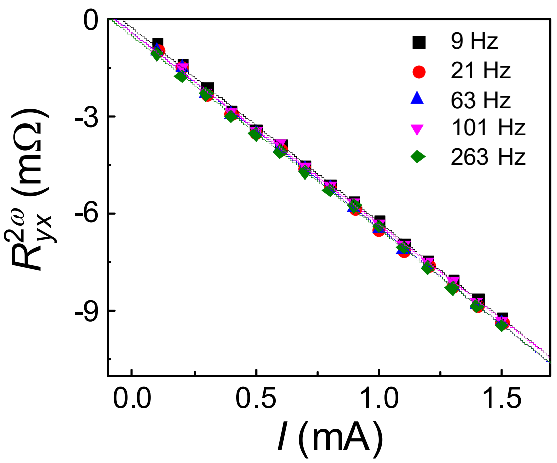

Supplementary Figure 1. Frequency dependence of nonlinear transport. The second harmonic transverse resistance $R_{yx}^{2\omega}$ versus ac current amplitude I measured at different ac frequencies under the conditions of T = 50 K and zero magnetic field, for 20 QL Bi_2Se_3 with the current along the $\overline{\Gamma}\overline{K}$ direction.

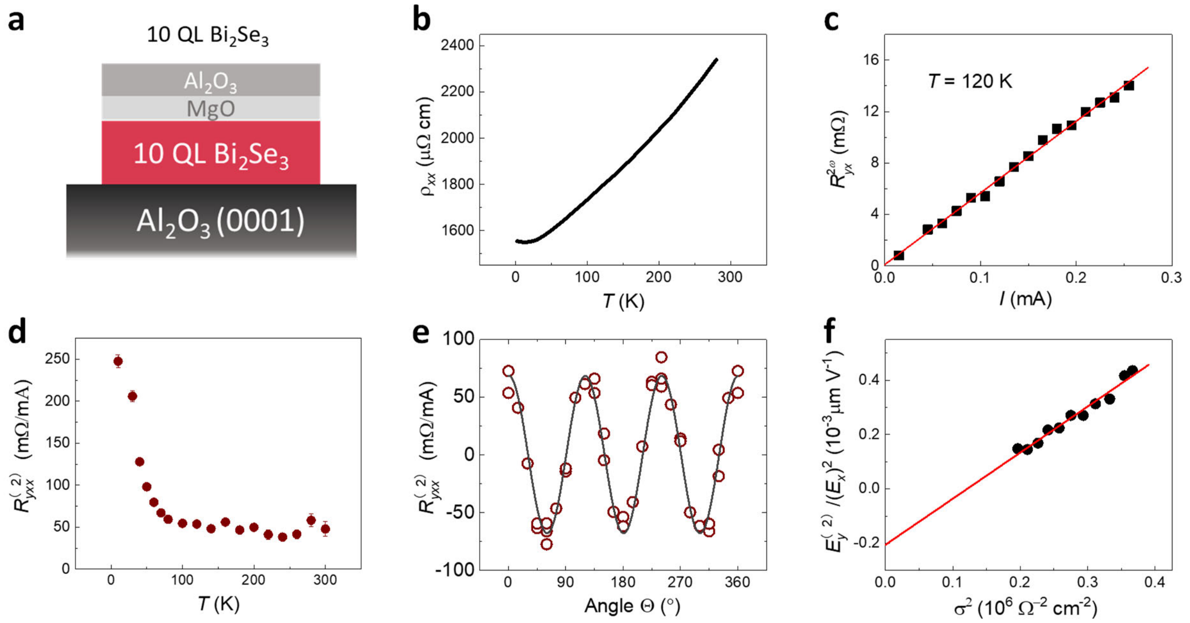

Supplementary Figure 2. Nonlinear transverse transport under zero magnetic field in 10 QL Bi_2Se_3. **a**, Schematic of the sample structure. **b**, Temperature dependence of the longitudinal resistivity (ρ_{xx}) under zero magnetic field. **c**, The second harmonic transverse resistance ($R_{yx}^{2\omega}$) versus current (I). The red line is a linear fit to the data. **d**, $R_{yxx}^{(2)}$ as a function of temperature. **e**, $R_{yxx}^{(2)}$ as a function of current direction, showing a three-fold symmetry. The data in panels **c**,**e** were collected at $T = 120$ K. **f**, $E_y^{(2)} / E_x^2$ versus the square of the longitudinal conductivity (σ^2). The red line is a linear fit to the experimental data.

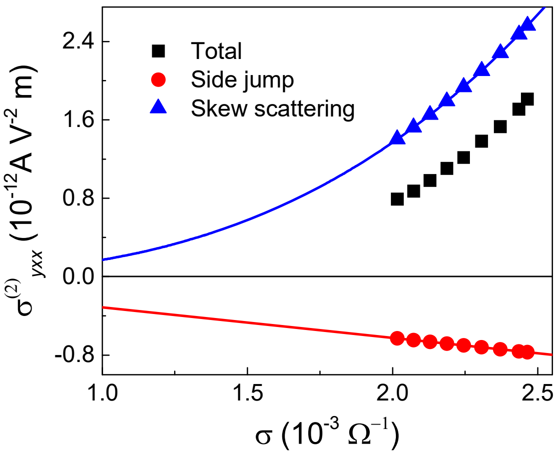

Supplementary Figure 3. The nonlinear transverse conductivity versus the longitudinal linear conductivity in 2-dimension. The total nonlinear conductivity (black) measured in our experiment is separated into the contribution of skew scattering (blue) and side jump (red) based on their different scaling with the linear conductivity in 20 QL Bi_2Se_3.

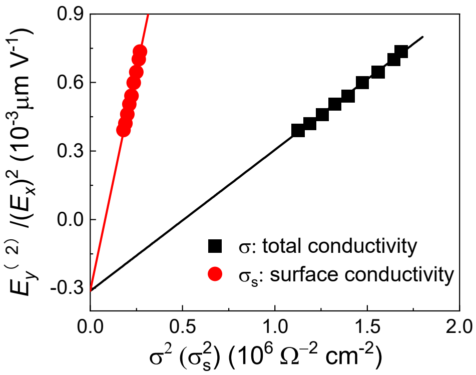

Supplementary Figure 4. $E_y^{(2)} / E_x^2$ **versus the square of the linear conductivity for the whole film (black curve) and the surface state (red curve) at the current angle Θ = 0°.** The lines are linear fits to the experimental data. We estimate that the surface state contributes to 40% of the total linear conductivity by assuming the carrier mobility are similar at surface and bulk states. The scaling with the total linear conductivity (black) evaluates the averaged nonlinear transverse response of the whole Bi_2Se_3 film, while the scaling with the surface linear conductivity (red) evaluates the nonlinear transverse response of the surface state. The linear slop *a* in the red fitting is 6 times larger than that in the black one, while the intercept *b* is almost the same for the two cases.

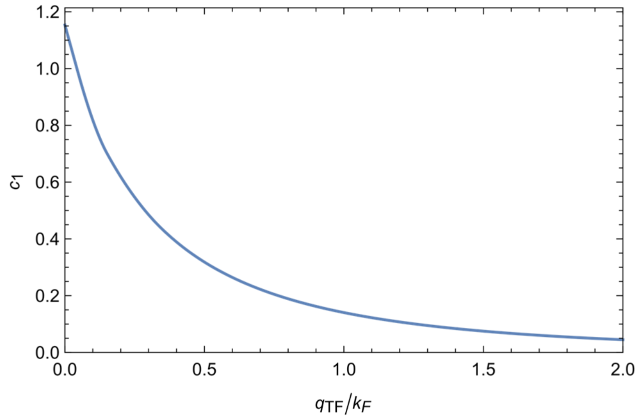

Supplementary Figure 5. Effect of the Thomas-Fermi screening to skew scattering. The coefficient $c_1\left(\frac{q_{\mathrm{TF}}}{k_F}\right)$ in the antisymmetric scattering rate $w_{\boldsymbol{kk}'}^{(A)}$ and hence the skew scattering time $\tilde{\tau}$ depends on the Thomas-Fermi wave vector q_{TF}. Skew scattering becomes weaker with stronger screening, i.e., large q_{TF}.

References

1. Beidenkopf, H., *et al.* Spatial fluctuations of helical Dirac fermions on the surface of topological insulators. *Nat. Phys.* **7**, 939-943 (2011).
2. Foster, M.S. Multifractal nature of the surface local density of states in three-dimensional topological insulators with magnetic and nonmagnetic disorder. *Phys. Rev. B* **85**, 085122 (2012).
3. Liu, Y., *et al.* Zeeman splitting and dynamical mass generation in Dirac semimetal $ZrTe_5$. *Nat. Commun.* **7**, 12516 (2016).
